# Literature-based discovery of diabetes- and ROS-related targets

**DOI:** 10.1186/1755-8794-3-49

**Published:** 2010-10-27

**Authors:** Junguk Hur, Kelli A Sullivan, Adam D Schuyler, Yu Hong, Manjusha Pande, David J States, H V Jagadish, Eva L Feldman

**Affiliations:** 1 Bioinformatics Program, University of Michigan, Ann Arbor, MI 48109, USA; 2 Department of Neurology, University of Michigan, Ann Arbor, MI 48109, USA; 3 National Center for Integrative Biomedical Informatics, University of Michigan, Ann Arbor, MI 48109, USA; 4 Department of Molecular, Microbial and Structural Biology, University of Connecticut Health Center, Farmington, CT 06030, USA; 5 School of Health Information Science, University of Texas, Houston, TX 77030, USA

## Abstract

**Background:**

Reactive oxygen species (ROS) are known mediators of cellular damage in multiple diseases including diabetic complications. Despite its importance, no comprehensive database is currently available for the genes associated with ROS.

**Methods:**

We present ROS- and diabetes-related targets (genes/proteins) collected from the biomedical literature through a text mining technology. A web-based literature mining tool, SciMiner, was applied to 1,154 biomedical papers indexed with diabetes and ROS by PubMed to identify relevant targets. Over-represented targets in the ROS-diabetes literature were obtained through comparisons against randomly selected literature. The expression levels of nine genes, selected from the top ranked ROS-diabetes set, were measured in the dorsal root ganglia (DRG) of diabetic and non-diabetic DBA/2J mice in order to evaluate the biological relevance of literature-derived targets in the pathogenesis of diabetic neuropathy.

**Results:**

SciMiner identified 1,026 ROS- and diabetes-related targets from the 1,154 biomedical papers (http://jdrf.neurology.med.umich.edu/ROSDiabetes/). Fifty-three targets were significantly over-represented in the ROS-diabetes literature compared to randomly selected literature. These over-represented targets included well-known members of the oxidative stress response including catalase, the NADPH oxidase family, and the superoxide dismutase family of proteins. Eight of the nine selected genes exhibited significant differential expression between diabetic and non-diabetic mice. For six genes, the direction of expression change in diabetes paralleled enhanced oxidative stress in the DRG.

**Conclusions:**

Literature mining compiled ROS-diabetes related targets from the biomedical literature and led us to evaluate the biological relevance of selected targets in the pathogenesis of diabetic neuropathy.

## Background

Diabetes is a metabolic disease in which the body does not produce or properly respond to insulin, a hormone required to convert carbohydrates into energy for daily life. According to the American Diabetes Association, 23.6 million children and adults, approximately 7.8% of the population in the United States, have diabetes [1]. The cost of diabetes in 2007 was estimated to be $174 billion [1]. The micro- and macro-vascular complications of diabetes are the most common causes of renal failure, blindness and amputations leading to significant mortality, morbidity and poor quality of life; however, incomplete understanding of the causes of diabetic complications hinders the development of mechanism-based therapies.

*In vivo *and *in vitro *experiments implicate a number of enzymatic and non-enzymatic metabolic pathways in the initiation and progression of diabetic complications [2] including: (1) increased polyol pathway activity leading to sorbitol and fructose accumulation, NAD(P)-redox imbalances and changes in signal transduction; (2) non-enzymatic glycation of proteins yielding "advanced glycation end-products" (AGEs); (3) activation of protein kinase C (PKC), initiating a cascade of intracellular stress responses; and (4) increased hexosamine pathway flux [2,3]. Only recently has a link among these pathways been established that provides a unified mechanism of tissue damage. Each of these pathways directly and indirectly leads to overproduction of reactive oxygen species (ROS) [2,3].

ROS are highly reactive ions or small molecules including oxygen ions, free radicals and peroxides, formed as natural byproducts of cellular energy metabolism. ROS are implicated in multiple cellular pathways such as mitogen-activated protein kinase (MAPK) signaling, c-Jun amino-terminal kinase (JNK), cell proliferation and apoptosis [4-6]. Due to the highly reactive properties of ROS, excessive ROS may cause significant damage to proteins, DNA, RNA and lipids. All cells express enzymes capable of neutralizing ROS. In addition to the maintenance of antioxidant systems such as glutathione and thioredoxins, primary sensory neurons express two main detoxifying enzymes: superoxide dismutase (SOD) [7] and catalase [8]. SOD converts superoxide (O_2_-) to H_2_O_2_, which is reduced to H_2_O by glutathione and catalase [8]. SOD1 is the main form of SOD in the cytoplasm; SOD2 is located within the mitochondria. In neurons, SOD1 activity represents approximately 90% of total SOD activity and SOD2 approximately 10% [9]. Under diabetic conditions, this protective mechanism is overwhelmed due to the substantial increase in ROS, leading to cellular damage and dysfunction [10].

The idea that increased ROS and oxidative stress contribute to the pathogenesis of diabetic complications has led scientists to investigate different oxidative stress pathways [7,11]. Inhibition of ROS or maintenance of euglycemia restores metabolic and vascular imbalances and blocks both the initiation and progression of complications [12,13]. Despite the significant implications and extensive research into the role of ROS in diabetes, no comprehensive database regarding ROS-related genes or proteins is currently available.

In the present study, a comprehensive list of ROS- and diabetes-related targets (genes/proteins) was compiled from the biomedical literature through text mining technology. SciMiner, a web-based literature mining tool [14], was used to retrieve and process documents and identify targets from the text. SciMiner provides a convenient web-based platform for target-identification within the biomedical literature, similar to other tools including EBIMed [15], ALI BABA [16], and PolySearch [17]; however, SciMiner is unique in that it searches full text documents, supports free-text PubMed query style, and allows the comparison of target lists from multiple queries.

The ROS-diabetes targets collected by SciMiner were further tested against randomly selected non-ROS-diabetes literature to identify targets that are significantly over-represented in the ROS-diabetes literature. Functional enrichment analyses were performed on these targets to identify significantly over-represented biological functions in terms of Gene Ontology (GO) terms and pathways.

In order to confirm the biological relevance of the over-represented ROS-diabetes targets, the gene expression levels of nine selected targets were measured in dorsal root ganglia (DRG) from mice with and without diabetes. DRG contain primary sensory neurons that relay information from the periphery to the central nervous system (CNS) [7,10,18]. Unlike the CNS, DRG are not protected by a blood-nerve barrier, and are consequently vulnerable to metabolic and toxic injury [19]. We hypothesize that differential expression of identified targets in DRG would confirm their involvement in the pathogenesis of diabetic neuropathy.

## Methods

### Defining ROS-diabetes literature

To retrieve the list of biomedical literature associated with ROS and diabetes, PubMed was queried using ("Reactive Oxygen Species"[MeSH] AND "Diabetes Mellitus"[MeSH]). This query yielded 1,154 articles as of April 27, 2009. SciMiner, a web-based literature mining tool [14], was used to retrieve and process the abstracts and available full text documents to identify targets (full text documents were available for approximately 40% of the 1,154 articles). SciMiner-identified targets, reported in the form of HGNC [HUGO (Human Genome Organization) Gene Nomenclature Committee] genes, were confirmed by manual review of the text.

### Comparison with human curated data (NCBI Gene2PubMed)

The NCBI Gene database provides links between Gene and PubMed. The links are the result of (1) manual curation within the NCBI via literature analysis as part of generating a Gene record, (2) integration of information from other public databases, and (3) GeneRIF (Gene Reference Into Function) in which human experts provide a brief summary of gene functions and make the connections between citation (PubMed) and Gene databases. For the 1,154 ROS-diabetes articles, gene-paper associations were retrieved from the NCBI Gene database. Non-human genes were mapped to homologous human genes through the NCBI HomoloGene database. The retrieved genes were compared against the SciMiner derived targets. Any genes missed by SciMiner were added to the ROS-diabetes target set.

### Protein-protein interactions among ROS-diabetes targets

To indirectly examine the association of literature derived targets (by SciMiner and NCBI Gene2PubMed) with ROS and diabetes, protein-protein interactions (PPIs) among the targets were surveyed. This was based on an assumption that targets are more likely to have PPIs with each other if they are truly associated within the same biological functions/pathways. A PPI network of the ROS-diabetes targets was generated using the Michigan Molecular Interactions (MiMI, http://mimi.ncibi.org/) database [20] and compared against 100 PPI networks of randomly drawn sets (the same number of the ROS-diabetes target set) from HUGO. A standard Z-test and one sample T-test were used to calculate the statistical significance of the ROS-diabetes PPI network with respect to the random PPI networks.

### Functional enrichment analysis

Literature derived ROS-diabetes targets (by SciMiner and NCBI Gene2PubMed) were subject to functional enrichment analyses to identify significantly over-represented biological functions in terms of Gene Ontology [21], pathways (Kyoto Encyclopedia of Genes and Genomes (KEGG, http://www.genome.jp/kegg/) [22] and Reactome http://www.reactome.org/[23]). Fisher's exact test [24] was used to calculate the statistical significance of these biological functions with Benjamini-Hochberg (BH) adjusted p-value < 0.05 [25] as the cut-off.

### Over-represented ROS-diabetes targets

#### Defining background corpora

To identify a subset of targets that are highly over-represented within the ROS-diabetes targets, the frequency of each target (defined as the number of documents in which the target was identified divided by the number of total documents in the query) was compared against the frequencies in randomly selected background corpora. Depending on how the background set is defined, over-represented targets may vary widely; therefore, to maintain the background corpora close to the ROS and diabetes context, documents were selected from the same journal, volume, and issue of the 1,154 ROS-diabetes documents, but were NOT indexed with "Reactive Oxygen Species"[MeSH] nor "Diabetes Mellitus"[MeSH]. For example, one of the ROS-diabetes articles (PMID: 18227068), was published in the Journal of Biological Chemistry, Volume 283, Issue 16. This issue contained 85 papers, 78 of which were not indexed with either "Reactive Oxygen Species"[MeSH] or "Diabetes Mellitus"[MeSH] indexed. One of these 78 papers was randomly selected as a background document. Three sets of 1,154 documents were selected using this approach and processed using SciMiner. Identified targets were confirmed by manual review for accuracy.

#### Identifying significantly over-represented targets

ROS-diabetes targets were tested for over-representation against targets identified from the three background sets. Fisher's exact test was used to determine if the frequency of each target in the ROS-diabetes target set was significantly different from that of the background sets. Any targets with a BH adjusted p-value < 0.05 in at least two of the three comparisons were deemed to be an over-represented ROS-diabetes target. Functional enrichment analyses were performed on these over-represented ROS-diabetes targets as described above.

#### Selecting targets for real-time RT-PCR

A subset of targets were selected for RT-PCR from the top 10 over-represented ROS-diabetes targets excluding insulin and NADPH oxidase 5 (NOX5), which does not have a mouse ortholog. Nitric oxide synthase 1 (NOS1), the main generator of nitric oxide, ranked at the 15^th ^position and was additionally selected for inclusion in the test set.

### Differential gene expression by real-time RT-PCR

#### Mice

DBA/2J mice were purchased from the Jackson Laboratory (Bar Harbor, ME). Mice were housed in a pathogen-free environment and cared for following the University of Michigan Committee on the Care and Use of Animals guidelines. Mice were fed AIN76A chow (Research Diets, New Brunswick, NJ). Male mice were used for this study.

#### Induction of diabetes

Two treatment groups were defined: control (n = 4) and diabetic (n = 4). Diabetes was induced at 13 weeks of age by low-dose streptozotocin (STZ) injections, 50 mg/kg/day for five consecutive days. All diabetic mice received LinBit sustained release insulin implants (LinShin, Toronto, Canada) at 8 weeks post-STZ treatment. Insulin implants were replaced every 4 weeks, at 12 and 16 weeks post-STZ treatment. At 20 weeks post-STZ treatment, mice were euthanized by sodium pentobarbital overdose and DRG were harvested as previously described [26].

#### Real-time RT-PCR

The gene expression of the selected nine literature-derived ROS-diabetes targets in DRG was measured using real-time RT-PCR in duplicate. The amount of mRNA isolated from each DRG was normalized to an endogenous reference [Tbp: TATA box binding protein; Δ cycle threshold (C_T_)].

## Results

### Identification of ROS-diabetes targets

A total of 1,021 unique targets were identified by SciMiner from the 1,154 ROS-diabetes papers defined by the query of ("Reactive Oxygen Species"[MeSH] AND "Diabetes Mellitus"[MeSH]) and confirmed by manual review. Table 1 contains the top 10 most frequently mentioned targets in the ROS-diabetes papers. Insulin was the most frequently mentioned target, followed by superoxide dismutase 1 and catalase.

**Table 1:** Top 10 most frequent ROS-diabetes targets

Symbol	Name	#Paper	Match Strings
INS	insulin	503	INS | insulin | proinsulin |
SOD1	superoxide dismutase 1	368	Sod1 | SOD1 | SOD1 | *
CAT	catalase	241	CAT | catalase | *
PRKCA	protein kinase C, alpha	194	PKCA | PKC-alpha | *
ALB	albumin	179	albumin | serum albumin |
NOX5	NADPH oxidase 5	177	NOX5 | nadph oxidase |
NOS2A	nitric oxide synthase 2A	144	NOS | iNOS | Nos2 |*
XDH	xanthine dehydrogenase	133	XOR |xanthine dehydrogenase| *
AGT	angiotensinogen	131	Ang-II | ANG | AGT | AngI | *
TNF	tumor necrosis factor	120	TNFA | TNF | TNF-alpha | *

* Matching strings are truncated to fit in the table. The full contents are available in the Additional File 2. '#Paper' refers to the number of documents in which each target was mentioned at least once.

The NCBI Gene2PubMed database, containing expert-curated associations between the NCBI Gene and PubMed databases, revealed 90 unique genes associated with the 1,154 ROS-diabetes papers (Additional File 1). SciMiner identified 85 out of these 90 targets, indicating a 94% recall rate. Five targets missed by SciMiner were added to the initial ROS-diabetes target set to result in 1,026 unique targets (Additional File 2).

### PPI network of the ROS-diabetes targets

**Figure 1:**
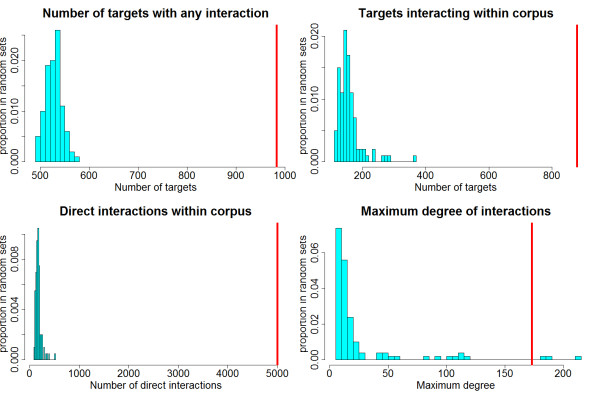
**Histograms of randomly generated PPI networks**. The histograms illustrate the distributions of 100 randomly generated networks, while the red line indicates the ROS-diabetes targets. The network of the ROS-diabetes targets is significantly different from the 100 randomly generated networks, indicating the overlap of ROS-diabetes targets with respect to the topic "Reactive Oxygen Species and Diabetes".

The PPI network among the ROS-diabetes targets was evaluated using MiMI interaction data. This was based on the assumption that targets commonly related to a certain topic are more likely to have frequent interactions with each other. One hundred PPI networks were generated for comparison using the same number of genes (1,026) randomly selected from the complete HUGO gene set (25,254). The PPI network of the ROS-diabetes targets was significantly different from the randomly generated networks indicating their strong association with the topic "ROS and Diabetes". Table 2 demonstrates that the mean number of targets with any PPI interaction in the randomly generated target sets was 528.9 (approximately 52% of 1,026 targets), while the number of targets with any PPI interaction in the ROS-diabetes target was 983 (96%). The number of targets interacting with each other was also significantly different between the random networks (mean = 155.4) and the ROS-diabetes network (mean = 879). Figure 1 illustrates the distributions of these measurements from the 100 random networks with the ROS-diabetes set depicted as a red vertical line. It is obvious that the PPI network of the ROS-diabetes targets is significantly different from the random networks.

**Table 2:** Summary of 100 randomly generated PPI networks

	# of targets with any interaction	# of targets interacting with each other	# of direct interactions among targets	Max degree*
ROS-diabetes Targets	983	879	5002	173

Mean (100 networks)	528.9	155.4	165.4	25
STDEV (100 networks)	16	36.2	54.2	39.7
Z-Score	28.5	20	89.2	3.7
P-value(Z)	0	0	0	9.60E-05
T-Statistics	-284.8	-200	-891.9	-37.3
P-value(T)	4.60E-146	6.70E-131	4.00E-195	4.20E-60

* Max degree refers to the number of interactions of the most highly interacting target.

### Functional enrichment analyses of the ROS-diabetes targets

Functional enrichment analyses of the 1,026 ROS-diabetes targets were performed to identify over-represented biological functions of the ROS-diabetes targets. After Benjamini-Hochberg correction, a total of 189 molecular functions, 450 biological processes, 73 cellular components and 341 pathways were significantly enriched in the ROS-diabetes targets when compared against all the HUGO genes (see Additional Files 3, 4, 5 and 6 for the full lists). Table 3 lists the top 3 most over-represented GO terms and pathways ranked by p-values of Fisher's exact test: e.g., apoptosis, oxidoreductase activity and insulin signaling pathway.

**Table 3:** Enriched functions of 1,026 ROS-diabetes targets

Category	Term	#target	p-value	Fold
Biological Processes GO	metabolic process	113	3.40E-26	3.3
	protein amino acid phosphorylation	98	2.90E-24	3.5
	response to hypoxia	36	8.80E-24	12

Molecular Functions GO	protein binding	514	2.80E-71	2.1
	oxidoreductase activity	103	1.50E-31	4.2
	transferase activity	148	1.70E-26	2.7

Cellular Components GO	cytoplasm	381	1.50E-57	2.3
	extracellular region	220	9.10E-44	2.9
	mitochondrion	154	6.30E-43	3.9

Pathway	Focal adhesion	75	2.40E-42	9.4
	Apoptosis	49	6.70E-35	14.5
	MAPK signaling pathway	73	4.30E-34	6.9

'#target' refers to the number of ROS-diabetes targets with each biological function with Benjamini-Hochberg adjusted p-values. Fold is the ratio of targets from the ROS-diabetes set to the complete HUGO gene set.

### Identification of over-represented ROS-diabetes targets

**Figure 2:**
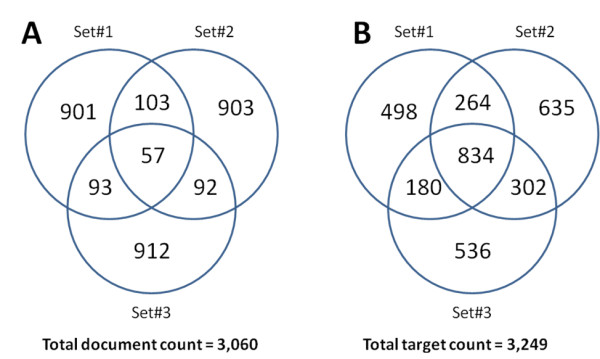
**Venn diagrams of document compositions and identified targets of the randomly generated background sets**. Approximately 90% of the selected background documents were unique to individual set (A), while 50% of the identified targets were identified in at least one of the three background document sets (B).

To identify the ROS-diabetes targets highly over-represented in ROS-diabetes literature, three sets of background corpora of the same size (n = 1,154 documents) were generated using the same journal, volume and issue approach. The overlap among the three background sets in terms of documents and identified targets are illustrated in Figure 2. Approximately 90% of the selected background documents were unique to the individual set, while 50% of the identified targets were identified in at least one of the three background document sets. The frequencies of the identified targets were compared among the background sets for significant differences. None of the targets had a BH adjusted p-value < 0.05, indicating no significant difference among the targets from the three different background sets (See Additional File 7).

Comparisons of the ROS-diabetes targets against these background sets revealed 53 highly over-represented ROS-diabetes targets as listed in Table 4. These 53 targets were significant (p-value < 0.05) against all three background sets and significant following Benjamini-Hochberg multiple testing correction (BH adjusted p-value < 0.05) against at least two of the three background sets. SOD1 was the most over-represented in the ROS-diabetes targets.

**Table 4:** 53 targets over-represented in ROS-diabetes literature

Rank	Symbol	HUGO_ID	Name	#Paper	BG #1	BG #2	BG #3
1	SOD1	11179	superoxide dismutase 1, soluble (amyotrophic lateral sclerosis 1 (adult))	368	3.1E-84	2.0E-78	2.0E-78
2	CAT	1516	catalase	241	2.1E-50	3.9E-44	3.9E-44
3	NOX5	14874	NADPH oxidase, EF-hand calcium binding domain 5	177	3.1E-42	3.6E-39	2.1E-37
4	INS	6081	insulin	503	5.9E-41	2.0E-43	2.3E-39
5	XDH	12805	xanthine dehydrogenase	133	1.5E-30	1.2E-28	8.8E-28
6	PRKCA	9393	protein kinase C, alpha	194	7.1E-23	6.4E-26	8.9E-24
7	NCF1	7660	neutrophil cytosolic factor 1, (chronic granulomatous disease, autosomal 1)	72	7.6E-19	7.7E-16	8.7E-16
8	NOS3	7876	nitric oxide synthase 3 (endothelial cell)	115	1.6E-18	3.9E-16	7.6E-18
9	SOD2	11180	superoxide dismutase 2, mitochondrial	85	2.1E-18	7.7E-16	3.8E-15
10	CYBA	2577	cytochrome b-245, alpha polypeptide	69	4.2E-17	5.0E-13	6.9E-14
11	NOS2A	7873	nitric oxide synthase 2A (inducible, hepatocytes)	144	3.9E-16	5.2E-12	4.5E-14
12	AGT	333	angiotensinogen (serpin peptidase inhibitor, clade A, member 8)	131	1.8E-14	1.4E-09	3.5E-08
13	AKR1B1	381	aldo-keto reductase family 1, member B1 (aldose reductase)	61	8.0E-13	9.5E-13	3.6E-11
14	CYBB	2578	cytochrome b-245, beta polypeptide (chronic granulomatous disease)	49	4.0E-12	2.6E-09	5.8E-11
15	NOS1	7872	nitric oxide synthase 1 (neuronal)	82	4.9E-12	3.7E-10	4.7E-09
16	NCF2	7661	neutrophil cytosolic factor 2 (65 kDa, chronic granulomatous disease, autosomal 2)	50	2.4E-11	1.5E-09	3.8E-08
17	CYCS	19986	cytochrome c, somatic	81	8.7E-10	2.2E-10	2.1E-10
18	HBB	4827	hemoglobin, beta	101	1.4E-08	5.9E-10	2.2E-08
19	GSR	4623	glutathione reductase	61	1.4E-08	4.8E-08	4.8E-08
20	UCP1	12517	uncoupling protein 1 (mitochondrial, proton carrier)	38	4.1E-07	2.1E-06	9.7E-06
21	NOX4	7891	NADPH oxidase 4	31	6.2E-07	2.3E-04	2.7E-05
22	PARP1	270	poly (ADP-ribose) polymerase family, member 1	37	7.1E-07	1.1E-07	5.3E-05
23	UCP2	12518	uncoupling protein 2 (mitochondrial, proton carrier)	34	7.0E-07	4.5E-06	2.1E-05
24	HBA1	4823	hemoglobin, alpha 1	30	1.1E-06	1.2E-06	9.3E-06
25	ALB	399	albumin	179	7.0E-06	4.9E-06	1.7E-06
26	NOX1	7889	NADPH oxidase 1	30	8.2E-06	8.6E-06	9.7E-06
27	NFKB1	7794	nuclear factor of kappa light polypeptide gene enhancer in B-cells 1 (p105)	90	9.4E-06	1.2E-04	4.5E-04
28	VEGFA	12680	vascular endothelial growth factor A	57	2.6E-04	1.9E-04	4.1E-03
29	SOD3	11181	superoxide dismutase 3, extracellular	18	2.5E-04	8.1E-02	3.4E-02
30	REN	9958	renin	51	3.6E-04	2.2E-02	7.2E-02
31	MPO	7218	myeloperoxidase	28	5.7E-04	2.4E-01	5.1E-02
32	SORD	11184	sorbitol dehydrogenase	15	1.8E-03	1.9E-03	1.8E-03
33	COL4A1	2202	collagen, type IV, alpha 1	15	1.8E-03	1.3E-02	1.8E-03
34	TGFA	11765	transforming growth factor, alpha	46	2.1E-03	3.5E-02	3.5E-04
35	ACE	2707	angiotensin I converting enzyme (peptidyl-dipeptidase A) 1	69	3.8E-03	1.1E-02	1.1E-02
36	AGTR1	336	angiotensin II receptor, type 1	36	3.7E-03	4.9E-02	1.8E-03
37	G6PD	4057	glucose-6-phosphate dehydrogenase	19	5.6E-03	3.7E-01	2.1E-01
38	CP	2295	ceruloplasmin (ferroxidase)	13	6.2E-03	3.1E-01	2.9E-01
39	NCF4	7662	neutrophil cytosolic factor 4, 40kDa	16	6.7E-03	9.9E-04	9.9E-04
40	MT-CYB	7427	mitochondrially encoded cytochrome b	15	1.3E-02	1.3E-02	1.3E-01
41	DUOX1	3062	dual oxidase 1	11	2.2E-02	2.9E-01	1.1E-01
42	SERPINE1	8583	serpin peptidase inhibitor, clade E (nexin, plasminogen activator inhibitor type 1), member 1	37	2.4E-02	2.5E-02	1.1E-03
43	GSTCD	25806	glutathione S-transferase, C-terminal domain containing	37	2.4E-02	3.8E-01	9.1E-02
44	COQ7	2244	coenzyme Q7 homolog, ubiquinone (yeast)	16	2.8E-02	1.9E-01	3.1E-02
45	RAC1	9801	ras-related C3 botulinum toxin substrate 1 (rho family, small GTP binding protein Rac1)	18	3.0E-02	4.3E-01	7.8E-02
46	MAOB	6834	monoamine oxidase B	10	3.9E-02	4.1E-01	4.4E-01
47	UCP3	12519	uncoupling protein 3 (mitochondrial, proton carrier)	17	4.7E-02	1.7E-02	1.8E-02
48	VCAM1	12663	vascular cell adhesion molecule 1	29	5.4E-02	6.3E-02	3.5E-02
49	AKT1	391	v-akt murine thymoma viral oncogene homolog 1	75	5.5E-02	4.9E-02	6.4E-02
50	LEPR	6554	leptin receptor	21	8.7E-02	3.1E-01	1.4E-02
51	EDN1	3176	endothelin 1	38	8.8E-02	3.8E-01	2.6E-02
52	COL1A1	2197	collagen, type I, alpha 1	84	8.7E-02	2.6E-02	1.7E-01
53	CCL2	10618	chemokine (C-C motif) ligand 2	38	2.0E-01	4.9E-02	1.0E-02

'#Paper' is the number of papers in ROS-diabetes corpus; BG#1, BG#2 and BG#3 are Benjamini-Hochberg adjusted p-values between ROS-diabetes targets and background sets.

### Functional enrichment analyses of the over-represented ROS-diabetes targets

Functional enrichment analyses of the 53 ROS-diabetes targets were performed to identify over-represented biological functions. Following Benjamini-Hochberg correction, a total of 65 molecular functions, 209 biological processes, 26 cellular components and 108 pathways were significantly over-represented when compared against all the HUGO genes (see Additional Files 8, 9, 10 and 11 for the full lists). Table 5 shows the top 3 most significantly over-represented GO terms and pathways ranked by p-values of Fisher's exact test. GO terms related to oxidative stress such as "superoxide metabolic process", "superoxide release", "electron carrier activity" and "mitochondrion" were highly over-represented in the 53 ROS-diabetes targets.

**Table 5:** Enriched functions of the 53 over-represented targets in diabetes

Category	Term	# target	p-value	Fold
Biological Processes GO	superoxide metabolic process	7	3.70E-15	303
	electron transport	13	1.50E-12	16
	superoxide release	5	4.20E-11	298

Molecular Functions GO	electron carrier activity	15	1.80E-17	27
	oxidoreductase activity	18	2.20E-16	14
	iron ion binding	15	4.20E-16	21

Cellular Components GO	mitochondrion	13	9.90E-08	6
	extracellular space	10	6.60E-07	8
	soluble fraction	7	3.20E-06	11

Pathway	Leukocyte transendothelial migration	9	6.40E-12	36
	Small cell lung cancer	7	1.00E-09	38
	Formation of Platelet plug	6	1.10E-08	41

'#target' refers to the number of ROS-diabetes targets with each biological function with Benjamini-Hochberg adjusted p-values. Fold is the ratio of targets from the ROS-diabetes set to the complete HUGO gene set.

### Gene expression change in diabetes

**Figure 3:**
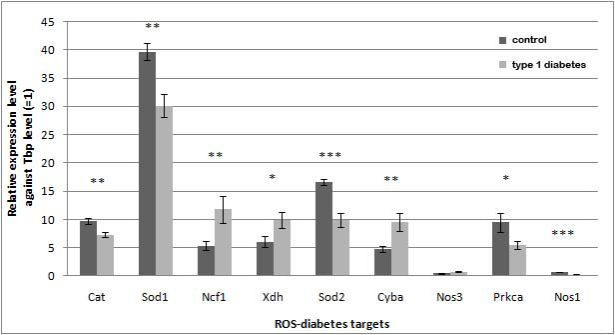
**Gene expression levels of selected ROS-diabetes targets in DRG examined by real-time RT-PCR**. Expression levels are relative to Tbp, an internal control (error bar = SEM) (*, p < 0.05; **, p < 0.01; ***, p < 0.001). Eight (Cat, Sod1, Ncf1, Xdh, Sod2, Cyba, Prkca, and Nos1) out of the nine selected ROS-diabetes genes were significantly regulated by diabetes.

Two groups of DBA/2J mice exhibited significantly different levels of glycosylated hemoglobin (%GHb). The mean ± SEM were 6.2 ± 0.3 for the non-diabetic control group and for 14.0 ± 0.8 for the diabetic group (p-value < 0.001), indicative of prolonged hyperglycemia in the diabetic group [26]. DRG were harvested from these animals for gene expression assays. Nine genes were selected from the top ranked ROS-diabetes targets: superoxide dismutase 1 (Sod1), catalase (Cat), xanthine dehydrogenase (Xdh), protein kinase C alpha (Prkca), neutrophil cytosolic factor 1 (Ncf1), nitric oxide synthase 3 (Nos3), superoxide dismutase 2 (Sod2), cytochrome b-245 alpha (Cyba), and nitric oxide synthase 1 (Nos1). Eight genes exhibited differential expression between diabetic and non-diabetic mice (p-value < 0.05) as shown in Figure 3. Cat, Sod1, Sod2, Prkca, and Nos1 expression levels were decreased, while Ncf1, Xdh, and Cyba expression levels were increased in diabetes.

## Discussion

Reactive oxygen species (ROS) are products of normal energy metabolism and play important roles in many other biological processes such as the immune response and signaling cascades [4-6]. As mediators of cellular damage, ROS are implicated in pathogenesis of multiple diseases including diabetic complications [27-30]. With the aid of literature mining technology, we collected 1,026 possible ROS-related targets from a set of biomedical literature indexed with both ROS and diabetes.

Fifty-three targets were significantly over-represented in the ROS-diabetes papers when compared against three background sets. Depending on how the background set is defined, the over-represented targets may vary widely. An ideal background set would be the entire PubMed set; however, this is not possible due to limited access to full texts and intense data processing. An alternative method would be to use only abstracts in PubMed, but this may not fully represent the literature. Using only the abstracts, our target identification method resulted in 21 (39%) of the 53 key ROS-diabetes targets (Additional File 12), suggesting the benefit of rich information in full text documents. In the present study, background documents were randomly selected from the same journal, volume, and issue of the 1,154 ROS-diabetes documents, which were not indexed with "Reactive Oxygen Species"[MeSH] nor "Diabetes Mellitus"[MeSH]. This approach maintained the background corpora not far from the ROS and diabetes context.

The gene expression levels of nine targets selected from the 53 over-represented ROS-diabetes targets were measured in diabetic and non-diabetic DRG. Our laboratory is particularly interested in deciphering the underlying mechanisms of diabetic neuropathy, a major complication of diabetes. Data published by our laboratory both *in vitro *and *in vivo *confirm the negative impact of oxidative stress in complication-prone neuron tissues like DRG [7,10,18,31]. In an effort to obtain diabetic neuropathy specific targets, SciMiner was employed to further analyze a subset of the ROS-diabetes papers (data not shown). Nerve growth factor (NGF) was identified as the most over-represented target in this subset when compared to the full ROS-diabetes set; however, NGF did not have statistical significance (BH adjusted p-value = 0.06). The relatively small numbers of papers and associated targets may have contributed to this non-significance. Therefore, the candidate targets for gene expression validation were selected from among the 53 over-represented ROS-diabetes targets derived from the full ROS-diabetes corpus.

Among the tested genes, the expression levels of Cat, Sod1, Sod2, Prkca, and Nos1 were decreased, while the expression levels of Ncf1, Xdh, and Cyba were increased under diabetic conditions. Cat, Sod1, and Sod2 are responsible for protecting cells from oxidative stress by destroying superoxides and hydrogen peroxides [8-11]. Decreased expression of these genes may result in oxidative stress [32]. Increased expression of Cyba and Ncf1, subunits of superoxide-generating nicotinamide adenine dinucleotide phosphate (NADPH) oxidase complex [30], also supports enhanced oxidative stress. Xdh and its inter-convertible form, Xanthine oxidase (Xod), showed increased activity in various rat tissues under oxidative stress conditions with diabetes [33], and also showed increased expression in diabetic DRG in the current study.

Unlike the above concordant genes, protein kinase C and nitric oxide synthases did not exhibit predicted expression changes in diabetes. Protein kinase C activates NADPH oxidase, further promoting oxidative stress in the cell [34,35]. Decreased expression of Prkca in our diabetic DRG is not parallel with expression levels of other enzymes expected to increase oxidative stress. Between the two nitric oxide synthases tested in the present study, Nos1 (neuronal) expression was significantly decreased (p-value < 0.001) in diabetes, while Nos3 (endothelial) expression was not significant (p-value = 0.06). The neuronal Nos1 is expected to play a major role in producing nitric oxide, another type of highly reactive free radical. Thus, with some exceptions, the majority of the differentially expressed genes in DRG show parallel results to the known activities of these targets in diabetes, suggesting enhanced oxidative stress in the diabetic DRG.

Assessment of antioxidant enzyme expression in diabetes has yielded a variety of results [36-40] depending upon the duration of diabetes, the tissue studied and other factors. In diabetic mice and rats, it is commonly reported that superoxide dismutases are down-regulated [37-40], where data regarding catalase are variable [36,40]. PKC is activated in diabetes, but most papers that examined mRNA demonstrated that its expression is largely unchanged [41].

Among the 53 over-represented ROS-diabetes targets, SOD1 was the most over-represented and was differentially expressed under diabetic and non-diabetic conditions. To the best of our knowledge, no published study has investigated the role of SOD1 in the onset and/or progression of diabetic neuropathy. Mutations of SOD1 have long been associated with the inherited form of amyotrophic lateral sclerosis (ALS) [42] and the theory of oxidative stress-based aging [43]. Early reports indicate that knockout of the SOD1 gene does not affect nervous system development [44], although recovery following injury is slow and incomplete [45,46]. With respect to diabetes, SOD1 KO accelerates the development of diabetic nephropathy [47] and cataract formation [48]. Thus, examining the SOD1 KO mouse as a model of diabetic neuropathy would be a reasonable follow-up study.

One limitation of the current approach using literature mining technology is incorrect or missed identification of the mentioned targets within the literature. Based on a performance evaluation using a standard text set BioCreAtIvE (Critical Assessment of Information Extraction systems in Biology) version 2 [49], SciMiner achieved 87.1% recall (percentage identification of targets in the given text), 71.3% precision (percentage accuracy of identified target) and 75.8% *F*-measure (harmonious average of recall and precision = (2 × recall × precision)/(recall + precision)) before manual revision [14]. In order to improve the accuracy of SciMiner's results, each target was manually reviewed and corrected by checking the sentences in which each target was identified. Approximately, 120 targets (~10% of the initially identified targets from the ROS-diabetes papers) were removed during the manual review process. The overall accuracy is expected to improve through the review process; however, the review process did not address targets missed by SciMiner, since we did not thoroughly review individual papers. Instead, 5 missed targets, whose associations with ROS-diabetes literature were available in the NCBI Gene2PubMed database, were added to the final ROS-diabetes target list (Additional File 2).

## Conclusions

The present approach enabled us to collect a comprehensive list of ROS and diabetes related targets and led us to confirm the biological relevance to diabetic neuropathy of the selected ROS-diabetes targets. Using SciMiner to identify significantly enriched targets is applicable to other disease topics of interest by providing a more focused subset of literature for review and by highlighting targets common to multiple manuscripts.

## List of abbreviations

NAD(P): nicotinamide adenine dinucleotide (phosphate); AGE: advanced glycoation end-products; PKC: protein kinase C; ROS: reactive oxygen species; MAPK: mitogen-activated protein kinase; JNK: c-Jun amino-terminal kinase; SOD1: superoxide dismutase 1; SOD2: superoxide dismutase 2; CAT: catalase; XDH: xanthine dehydrogenase; NCF1: neutrophil cytosolic factor 1; NOS3: nitric oxide synthase 3; CYBA: cytochrome b-245 alpha; NOS1: nitric oxide synthase 1; ALS: amyotrophic lateral sclerosis; BioCreAtIvE: Critical Assessment of Information Extraction systems in Biology; MiMI: Michigan Molecular Interactions; KEGG: Kyoto Encyclopedia of Genes and Genomes; STZ: streptozotocin; GO: gene ontology; DRG: dorsal root ganglia; CNS: central nervous system; HGNC: HUGO (human genome organization) nomenclature committee; PPI: protein-protein interaction; SEM: standard error mean.

## Competing interests

The authors declare that they have no competing interests.

## Authors' contributions

JH participated in the study design, performed the literature mining and functional enrichment analyses, and drafted the manuscript. KAS participated in the study design and drafted the manuscript. ADS participated in the statistical analysis. YH carried out the quantitative RT-PCR assay. MP participated in the manuscript revision. DJS participated in the study design and manuscript revision. HVJ participated in the study design and manuscript revision. ELF participated in the study design and manuscript revision. All authors read and approved the final manuscript.

## Pre-publication history

The pre-publication history for this paper can be accessed here:

http://www.biomedcentral.com/1755-8794/3/49/prepub

## Supplementary Material

Additional file 1 **The list of 90 genes from the NCBI Gene2PubMed database for the ROS-Diabetes literature (1,154 papers)**. Click here for file

Additional file 2 **The list of 1,026 ROS-Diabetes targets**. Click here for file

Additional file 3 **The enriched Molecular Functions Gene Ontology Terms in the 1,026 ROS-Diabetes targets**. Click here for file

Additional file 4 **The enriched Biological Processes Gene Ontology Terms in the 1,026 ROS-Diabetes targets**. Click here for file

Additional file 5 **The enriched Cellular Components Gene Ontology Terms in the 1,026 ROS-Diabetes targets**. Click here for file

Additional file 6 **The enriched pathways in the 1,026 ROS-Diabetes targets**. Click here for file

Additional file 7 **Comparisons of target frequencies among three background sets**. Click here for file

Additional file 8 **The enriched Molecular Functions Gene Ontology Terms in the Over-represented 53 ROS-Diabetes targets**. Click here for file

Additional file 9 **The enriched Biological Processes Gene Ontology Terms in the Over-represented 53 ROS-Diabetes targets**. Click here for file

Additional file 10 **The enriched Cellular Components Gene Ontology Terms in the Over-represented 53 ROS-Diabetes targets**. Click here for file

Additional file 11 **The enriched pathways in the Over-represented 53 ROS-Diabetes targets**. Click here for file

Additional file 12 **The Key 53 ROS-Diabetes Targets Identifiable Using Only the Abstracts**. Click here for file
